# The Importance of Synchrony and Temporal Order of Visual and Tactile Input for Illusory Limb Ownership Experiences – An fMRI Study Applying Virtual Reality

**DOI:** 10.1371/journal.pone.0087013

**Published:** 2014-01-31

**Authors:** Robin Bekrater-Bodmann, Jens Foell, Martin Diers, Sandra Kamping, Mariela Rance, Pinar Kirsch, Jörg Trojan, Xaver Fuchs, Felix Bach, Hüseyin Kemal Çakmak, Heiko Maaß, Herta Flor

**Affiliations:** 1 Department of Cognitive and Clinical Neuroscience, Central Institute of Mental Health, Medical Faculty Mannheim, Heidelberg University, Mannheim, Germany; 2 Department of Psychology, Florida State University, Tallahassee, Florida, United States of America; 3 Department of Psychology, University of Koblenz-Landau, Landau, Germany; 4 Institute for Applied Computer Science, Karlsruhe Institute of Technology, Eggenstein-Leopoldshafen, Germany; Royal Holloway, University of London, United Kingdom

## Abstract

In the so-called rubber hand illusion, synchronous visuotactile stimulation of a visible rubber hand together with one's own hidden hand elicits ownership experiences for the artificial limb. Recently, advanced virtual reality setups were developed to induce a virtual hand illusion (VHI). Here, we present functional imaging data from a sample of 25 healthy participants using a new device to induce the VHI in the environment of a magnetic resonance imaging (MRI) system. In order to evaluate the neuronal robustness of the illusion, we varied the degree of synchrony between visual and tactile events in five steps: in two conditions, the tactile stimulation was applied prior to visual stimulation (asynchrony of −300 ms or −600 ms), whereas in another two conditions, the tactile stimulation was applied after visual stimulation (asynchrony of +300 ms or +600 ms). In the fifth condition, tactile and visual stimulation was applied synchronously. On a subjective level, the VHI was successfully induced by synchronous visuotactile stimulation. Asynchronies between visual and tactile input of ±300 ms did not significantly diminish the vividness of illusion, whereas asynchronies of ±600 ms did. The temporal order of visual and tactile stimulation had no effect on VHI vividness. Conjunction analyses of functional MRI data across all conditions revealed significant activation in bilateral ventral premotor cortex (PMv). Further characteristic activation patterns included bilateral activity in the motion-sensitive medial superior temporal area as well as in the bilateral Rolandic operculum, suggesting their involvement in the processing of bodily awareness through the integration of visual and tactile events. A comparison of the VHI-inducing conditions with asynchronous control conditions of ±600 ms yielded significant PMv activity only contralateral to the stimulation site. These results underline the temporal limits of the induction of limb ownership related to multisensory body-related input.

## Introduction

The experience of body ownership (i.e., the subjective certainty that a body part belongs to oneself [1]) is an important feature of everyday perception. For more than ten years, the rubber hand illusion (RHI) has offered an opportunity to systematically manipulate the sense of body ownership [2]. In this paradigm, synchronous visuotactile stimulation of an observed rubber hand together with one's hidden hand leads to a perception of the rubber hand as belonging to one's own body. In a functional magnetic resonance imaging (fMRI) study, Ehrsson et al. [3] showed that bilateral activity in ventral premotor cortex (PMv) is directly associated with illusory ownership of the artificial hand. Damage in fibers connecting the contralateral PMv with other brain regions impairs the occurrence of RHI experiences [4], indicating its important role in the integration of multimodal sensory input as a prerequisite for experiencing illusory ownership. Together with intraparietal cortex (IPC), the PMv is suggested to code for the recalibration of the hand-centered coordinate systems of the body within its peripersonal space [5].

Synchrony between visual and tactile stimuli is a crucial feature in the RHI paradigm. While synchronous visuotactile input elicits experiences of illusory ownership in most of the participants, asynchronous stimulation prevents the occurrence of illusory sensations (e.g., [2], [3], [6], [7]). Asynchronies between visual and tactile stimulation of more than 300 ms significantly diminish the intensity of RHI sensations [7], indicating that this delay reflects the temporal limit for visuotactile integration. This matches other results for the consequences of temporal delays for the integration of body-related multisensory input [8], [9], [10].

Recently, advanced setups for RHI induction were described. Slater et al. [11] developed a virtual reality (VR) device to successfully induce a virtual hand illusion (VHI) by the application of synchronous visuotactile stimulation. In other VR setups, correlated sensorimotor input elicited the experience of body ownership [12], [13], [14], and multisensory stimulation is able to induce transformations of the perceived body [15], [16], [17], indicating that VR devices are appropriate for the examination of the processes involved in body ownership experiences. Since these setups allow for standardized conditions and for the application of standardized visual and tactile stimulation, they provide tighter control over the experimental manipulation of body ownership than other setups, in which the stimulation is manually applied by the experimenter. Consequently, VR devices are powerful tools to investigate the brain regions underlying body perception, especially by using imaging techniques with high spatial resolution such as functional magnetic resonance imaging (fMRI). We [18] recently introduced a VR device suitable for the analysis of the neuronal correlates of illusory ownership experiences in the environment of a magnetic resonance imaging system. In this setup, a virtual image of a hand is touched visually by a moving rod, while the real hand is stimulated using a pneumatically driven tactor. The software permits the control of several factors such as the stimulation site and the degree of synchrony between visual and tactile stimulation.

In the present study, we used this new VR device to systematically manipulate the degree of synchrony between visual and tactile events as well as their temporal order. The stimuli were applied to the participants' hidden left hand and a left hand in VR. We present subjective and neuronal data obtained in a sample of healthy participants to evaluate the suitability of the device in the MR scanner and the importance of such manipulations for body ownership processing.

## Materials and Methods

### Participants

We included 25 healthy participants (16 female), mostly composed of members of the Universities of Mannheim and Heidelberg. The average age was 29.00 years (SD = 6.83; range: 19–52). Since the hand presented in our VR setup had a skin color corresponding to a Caucasian ethnicity, we included only Caucasian participants. All of them were naïve about the purpose of the experiment. None of the participants reported a history of drug abuse or neurological or mental disorder. Left-handed participants (as assessed with the Edinburgh Handedness Inventory [19]) were excluded prior to the experiment. All participants had normal or corrected-to-normal vision. The study was approved by the ethics review board of the Medical Faculty Mannheim, Heidelberg University, and adhered to the Declaration of Helsinki. All participants gave written informed consent prior to taking part in the study.

### Virtual hand illusion device

**Figure 1:**
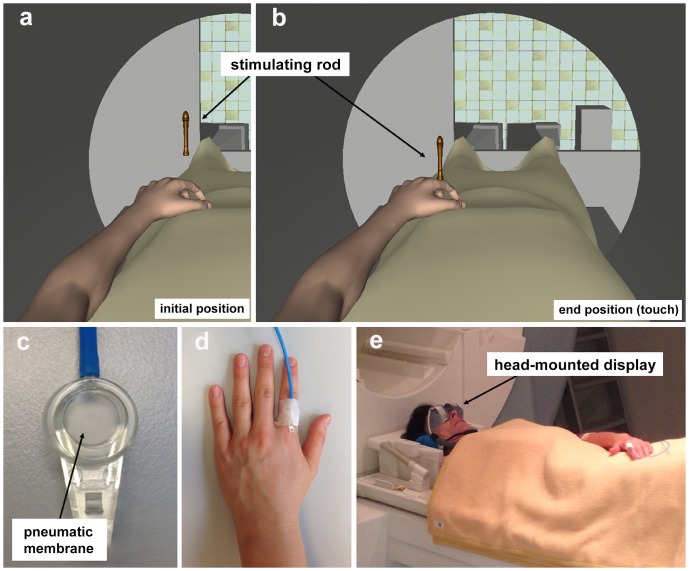
Setup of the virtual hand illusion. a) participant's view of the virtual reality environment, including the virtual limb and the stimulating rod at its starting position, b) the same view and rod at its end position touching the virtual finger, c) close-up picture of the pneumatic tactor, d) pneumatic tactor, attached to the participants' left index finger, e) participant lying on the table connected with the magnetic resonance imaging scanner. The participant has given written informed consent, as outlined in the PLOS consent form, to publication of her photograph.

The VHI was implemented using a VR device based on the simulation software KISMET (Kinematic Simulation, Monitoring and Off-Line Programming Environment for Telerobotics, V6.0.3, Karlsruhe, Germany), which was used to visualize the environment of an MRI (for technical details see [18], [20]). In this setting, a life-like model of an arm (i.e., hand, forearm, and parts of the upper arm) and a lower body covered by a blanket were modeled, simulating the egocentric perspective of a participant lying in an MRI scanner (Figure 1a and 1b). To achieve the desired posture for the limb model, the skeletal animation technique [21], [22] was used to deform a static geometry mesh. In addition, a virtual rod was designed as a kinematic object which can perform controlled vertical movements. This rod served as visual stimulator of the virtual hand, seemingly applying a localized touch to it. The location of the rod model can be freely arranged in 3D space in the virtual environment. By using a graphical user interface (GUI), several features of the VR setup can be modified, such as the selection of a left or a right arm model or the characteristics of the movement of the virtual rod in terms of movement amplitude, movement speed (up and down), and duration of pausing of the rod after descent. Additionally, the GUI allows varying the degree of synchrony between visual stimulation of the virtual hand and tactile stimulation of the participant's hand.

Tactile stimuli were presented to the participant's hand with a pneumatically driven tactor (a pneumatic finger clip, MEG International Services Ltd., Coquitlam, Canada; Figure 1c). These clips can be fixated at any position of the participant's target hand (i.e., the hand on which the VHI is to be induced) by using medical tapes. We used one clip attached at the top of the proximal phalanx of the index finger, matching the position of the virtual rod placed above this location (Figure 1d).

Via a pneumatic tube, the clips were connected with a custom-made pneumatic relay device (using elements available from Festo AG & Co. KG, Esslingen, Germany). This device was linked to the computer executing the KISMET software, which delivered signals for triggering the pneumatic stimulation. The pneumatic tubes were led out of the scanner room through underground cable shafts and connected to the relay device in the control room. The pressure of compressed air driving the pneumatic stimulation was set to 3 bar, which caused a clearly perceptible, but non-painful tactile stimulus on the stimulation site.

### VHI procedure in the fMRI scanner

The participant was instructed about the experimental procedure and the duration of the investigation, before he or she was positioned in the MR scanner (Figure 1e). As in previous studies (e.g., [2], [23]), we induced illusory ownership on the participant's left hand. The participants' right arm was placed under a blanket covering the body, whereas the left arm was situated on the blanket in a position identical to the virtual arm as shown in Figure 1a. The pneumatic clip was attached to the proximal phalanx of the participants' left index finger using medical tape. The functioning of the clip was tested by applying a single stimulus. The participant wore MR-compatible goggles (VisuaStimDigital, Resonance Technology, Inc., Northridge, CA, USA), displaying the KISMET graphical output in a double-monocular fashion. In order to align the perspective in VR with the participant's real body, the participant was asked to pull his or her chin slightly to the chest, simulating direct vision of the virtual limb. The participants were then instructed to observe the virtual hand during the entire session.

In RHI pre-tests on six individuals (using a modified paradigm already described by Bekrater-Bodmann et al. [23]), we found that localized touches induced vivid RHI experiences (mean value of the RHI vividness score, as calculated as described below, *M* = 3.97, SD = 1.86), comparable to that induced by stroking (*M* = 4.44, SD = 1.76), as long as the visuotactile stimulation was applied synchronously. In our VHI setup, both the visual stimulation in VR as well as the tactile stimulation through the pneumatic relay device was synchronized with the software running the MRI scanner. Although both the onset of the visual (start of the movement of the virtual rod) and the tactile stimulation (release of the compressed air) were triggered simultaneously, we had to adjust three parameters of the virtual rod (velocity and distance of down-movement as well as the starting point of the rod in virtual space) to account for perceived temporal incongruencies of visuotactile stimulation caused by the transportation of air along the pneumatic tube length of approximately seven meters. Thus, in order to align the visual with tactile stimulation in our VHI device, we surveyed five healthy participants prior to the main study. We varied the parameters of temporal characteristics of the virtual rod until the majority of participants perceived the seen and felt touch as simultaneous. These parameters served as the synchronous, or ‘0’ condition (meaning that there was a perceived asynchrony of 0 ms) and as the base from which temporal delays were determined.

**Figure 2:**
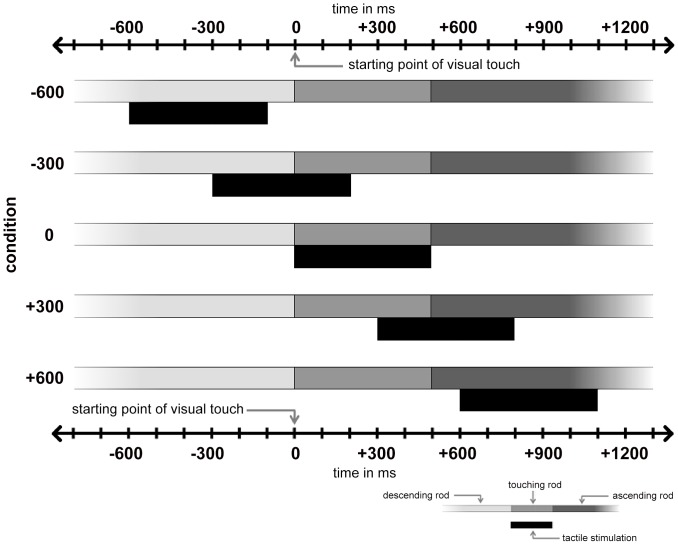
Design of the study. Displayed is the tactile stimulation in relation to the visual event for each condition. The 0 condition reflects the visuotactile stimulation in synchrony. Negative signs indicate the temporal delay (in milliseconds, ms), meaning that the tactile stimulation was applied prior to the visual stimulation; positive signs indicate that the tactile stimulation was applied after the visual stimulation.

We implemented five conditions in the fMRI experiment, varying the degree of synchrony and the temporal order of visual and tactile stimulation. In addition to the 0 condition, we chose values to operationalize four different asynchronous conditions. For setting the delays, we were guided by previous results revealing the temporal limits of synchrony between visual and tactile stimulation in the RHI paradigm: up to a delay of 300 ms between visual and tactile input, no reduction in illusion intensity is reported, whereas delays of 600 ms significantly reduce illusion intensity [7]. Consequently, we selected identical delays, operationalizing slight (300 ms) and distinct asynchrony (600 ms) between visual and tactile input. In order to examine the importance of temporal order of visual and tactile stimulation for the experience of illusory limb ownership, we varied the sequence of both modalities. In two conditions, the tactile stimulation was applied prior to the visual stimulation (‘−600’ and ‘−300’ conditions), and in another two conditions, the tactile stimulation was applied after the visual touch in VR (‘+600’ and ‘+300’ conditions). The design of the study is summarized in Figure 2.

Each condition was implemented in a separate scanning trial with a duration of 4∶34 min. We used a block design with blocks of 6 images ( = 20 s) of visuotactile stimulation (on-blocks), interspersed with 5 blocks of 7 images ( = 23.3 s) of rest (off-blocks). The simulation model of the moving rod was synchronized to the image recordings of the MRI. Each image of the on-block triggered the rod to move downwards and upwards and one single pneumatic stimulus was applied. To account for irregularities in the hemodynamic responses triggered by the visuotactile stimulation, we added a temporal jitter, with a randomized time delay of 0, 100, 200, 300, 400, or 500 ms before stimulation onset.

The sequence of conditions was randomized. After each trial, the vividness of the VHI experience was assessed using a modified version of a questionnaire introduced by Botvinick and Cohen [2], consisting of three items aiming at illusory limb ownership (see Table 1, targets; items # 1–3) and six items aiming at sensations not indicating illusory limb ownership or indicating suggestibility (see Table 2, distractors; items # 4–9). The sequence of items was randomized. The items were answered verbally using a discrete numerical scale ranging from 0 (not at all) to 10 (most intense). To create a sum score indicating an adjusted measure of the general vividness of the illusion, we subtracted the mean value of the six distractor items from the mean value of the three target items (*VHI vividness score*). Thus, positive scores indicate perceived ownership of the virtual hand, whereas negative scores represent a tendency to respond to distractor items (cf., [23]).

**Table 1:** Description of the items used to assess the virtual hand illusion. Bold font indicates target items.

Item #	Wording
**1**	**It seemed as if I were feeling the touch in the location where I saw the virtual hand touched.**
**2**	**I felt as if the virtual hand were my hand.**
**3**	**The touching of the virtual hand felt just like an actual touch.**
4	It felt as if my own hand had moved involuntarily.
5	It visually appeared as if the virtual hand had moved.
6	It seemed as if the touch I was feeling came from somewhere between my own hand and the virtual hand.
7	It seemed as if I might have more than two hands.
8	The virtual hand began to resemble my real hand, in terms of shape, skin tone, freckles or some other visual features.
9	My own hand felt artificial.

**Table 2:** Results for the conjunction analysis across all conditions.

Area	Brodmann Area	Hemisphere	Coordinate			Peak Z	Cluster size (in voxel)
			x	y	z		
Middle temporal gyrus	21/37	L	−44	−66	8	6.38**	246**
		L	−50	−72	4	6.12**	
Middle temporal gyrus	21/37	R	46	−64	4	6.45**	186**
Superior temporal gyrus	40/41	L	−54	−30	20	5.11*	33**
Supramarginal gyrus		L	−58	−22	22	5.54*	
Superior temporal gyrus	40/41	R	62	−30	22	6.27**	253**
Supramarginal gyrus		R	50	−26	24	5.44*	
Precentral gyrus	6	L	−44	−2	52	5.68*	16*
Middle occipital gyrus	18	L	−32	−94	−6	5.31*	12*

Coordinates in MNI space. L =  left; R =  right.

** *p*<.001 (FWE-corrected) *p<.05 (FWE-corrected).

### Acquisition of fMRI data

The fMRI scans were acquired with a MAGNETOM TRIO 3 T scanner (Siemens AG, Erlangen, Germany) using echo-planar imaging (EPI) with a matrix of 64×64 (TE = 45 ms, TR = 3300 ms) and 40 slices of 2.3 mm thickness (voxel size: 2.3 mm^3^, field of view: 220 mm) angulated in parallel to the AC-PC line, adjusted to include all frontal, central, parietal, temporal, and occipital cortical areas as well as upper parts of the cerebellum. Eighty whole-brain scans including six blocks of visuotactile stimulation with six scans each interspersed with five blocks without visuotactile stimulation of seven scans each were gathered per condition. The first three volumes of each trial were excluded prior to data analysis to allow for signal stability following onset transients. For anatomical reference, a T1-weighted anatomical data set (MPRAGE; slice thickness: 1.1 mm, TE = 2.98 ms, TR = 2300 ms, flip angle: 9°) was obtained.

### Analyses of rating data

The VHI vividness score is calculated by subtracting the mean of the distractor items from the mean of the target items, resulting in a score ranging from −10 to +10. Significant positive values would indicate that the illusion was induced. Therefore, we performed one-sample t-tests for the VHI vividness score in each condition with a test value of 0 and adjusted for multiple comparisons using Bonferroni-correction. Ratings across conditions were compared using an ANOVA for repeated measurements. One-tailed t-tests for paired samples were post-hoc applied to compare the VHI vividness scores in the asynchronous conditions with the synchronous condition. Further, we tested post-hoc for differences in VHI vividness scores related to the temporal order of visual and tactile stimulation (two-tailed). To account for alpha inflation due to multiple testing of a single hypothesis, we also adjusted these results applying Bonferroni-correction, if necessary. Statistical analyses were performed across the whole sample, VHI perceivers as well as VHI non-perceivers.

### Analyses of fMRI data

fMRI data were evaluated with Statistical Parametric Mapping software (SPM8; Wellcome Institute of Imaging Neuroscience, London, UK) implemented in Matlab 7.1 (Mathworks Inc., Natick, MA, USA). The data were realigned, corrected for slice timing effects, co-registered with a mean image of realigned and slice-timed images, applying the anatomical data as reference, normalized to the Montreal Neurological Institute (MNI) template and smoothed with a Gaussian kernel of 8 mm^3^ (full-width at half-maximum). The general linear model was estimated and included individual movement regressors as regressors of no interest.

First, we performed a whole brain regression for illusory ownership experiences (target item # 2) for the synchronous 0 condition, since this condition was expected to induce most vivid illusion experiences. We then performed a full factorial analysis for all conditions to identify significant brain activations involved in visuotactile integration. The factorial matrix resulted in a 1×5 design with five dependent levels for the factor *condition* (−600, −300, 0, +300, +600). We performed a conjunction analysis across all conditions to reveal brain regions with shared activation. Further, we contrasted conditions to obtain brain activity associated with illusory ownership experiences. Since we expected a significant reduction in the illusion vividness ratings only for distinct temporal asynchrony compared to synchrony [7], we contrasted the condition assumed to induce a VHI (i.e., the 0 condition) with the combined ±300 and ±600 conditions and vice versa. Finally, we contrasted the neuronal activity in conditions in which the tactile stimulation was applied prior to visual stimulation (−600/−300) with conditions in which the tactile stimulation was applied after visual stimulation (+600/+300) and vice versa in order to evaluate the importance of temporal order of visual and tactile input. The results of these contrasts as well as the conjunction analysis and the regression analysis are given at a threshold for whole brain analyses of *p*<.05, family-wise error (FWE) corrected for peak activity, with a cluster threshold of *k*>10 voxels.

Due to the importance of the PMv [3], [4], [24] and IPC [3], [25] for illusory ownership experiences, we specifically explored these regions in all analyses, in which they were not detected in the whole brain analysis, using a region of interest (ROI) approach. For the PMv, we used a specially created ROI based on previous results on visuotactile integration in body perception [3], [25], [26], [27], which was defined by interpolating and merging the peak coordinates reported in the literature to obtain an oblong volume covering the ventral parts of the premotor cortex associated with processing of the RHI. The diameter of this volume was adapted to the standard deviation of the single peaks. The left-hemispheric PMv ROI had a volume of *k* = 664 voxels and the right-hemispheric PMv ROI had a volume of *k* = 620 voxels. This ROI was previously used by Bekrater-Bodmann et al. [23]. For the IPC, we also used a special ROI likewise created on the basis of previous findings regarding body-related multisensory stimulation [3], [25], [27], [28], [29], [30] (volume of the right-hemispheric IPC ROI: 618 voxels; volume of the left-hemispheric IPC ROI: 574 voxels). Due to the strong a priori hypotheses about an involvement of these regions in the present experiments, the ROI analysis results are displayed with a threshold of *p*<.01, uncorrected for peak activity, and a cluster threshold of *k*>10 voxels. All these statistical analyses were performed across the whole sample, VHI perceivers as well as VHI non-perceivers.

## Results

### Results of rating data analyses

**Figure 3:**
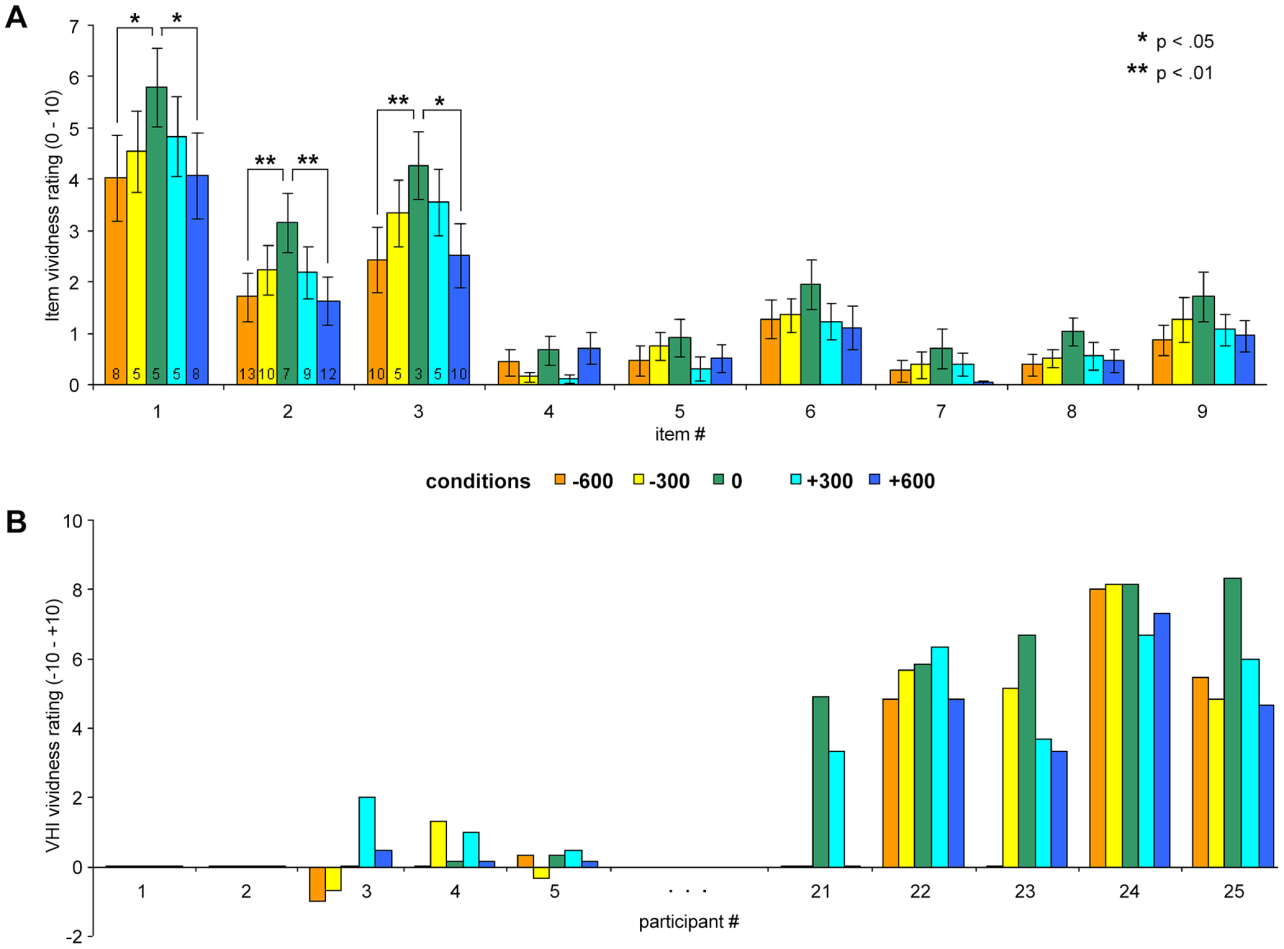
Illusion vividness ratings. a) Depicted are the virtual hand illusion (VHI) intensity ratings for each item in each condition. Items # 1–3 represent targets indicating ownership over the virtual hand, and items # 4–9 represent distractors. The number at the base of each target item bar indicates the number of non-responders (i.e., responses  = 0; only given for target items). Asterisks indicate significant differences between the synchronous and distinctly asynchronous conditions (Bonferroni-corrected p-values on item level). Note that there were only significant results for the target items. There were no significant differences between the synchronous and slightly asynchronous conditions for any item.Error bars indicate standard error; b) For illustrative purposes, the ratings of participants who responded minimally (participant # 1–5) or maximally (participant # 21–25) to illusion induction are depicted, arranged according to the proneness to perceive the VHI in the 0 condition (VHI vividness score). Colors indicate the conditions. The 0 condition reflects the visuotactile stimulation in synchrony. Negative signs indicate the temporal delay (in milliseconds), which means that the tactile stimulation was applied prior to the visual stimulation; positive signs indicate that the tactile stimulation was applied after the visual stimulation.

A number of participants in each condition did not perceive the VHI at all, which is indicated by negative or zero values in the VHI vividness scores; however, in the synchronous 0 condition as well as the slightly incongruent +300 condition we observed the smallest numbers of VHI non-perceivers (‘−600’: n = 8; ‘−300’: n = 6; ‘0’: n = 3; ‘+300’: n = 3; ‘+600’: n = 5). Ratings for the single items in each condition across all participants are given in Figure 3a. Figure 3b compares the individual VHI intensity ratings of the five participants who responded minimally or maximally to the illusion-indicating items.

In each condition, the target items were rated significantly higher than the distractor items, which is indicated by mean values for the VHI vividness score which were significantly different from 0 (‘−600’: *M* = 2.10, SD = 2.58; ‘−300’: *M* = 2.67, SD = 2.51; ‘0’: *M* = 3.23, SD = 2.58; ‘+300’: *M* = 2.87, SD = 2.55; ‘+600’: *M* = 2.11, SD = 2.50; 24 degrees of freedom, *t*-values ranging from 4.08 to 6.26; all *p*<.01). Afterwards, we performed an ANOVA for repeated measurements. Mauchly’s test indicated that the assumption of sphericity had been violated for the effect of condition, *χ* ^2^_9_ = 20.60, *p*<.05. Therefore, degrees of freedom were corrected using Greenhouse-Geisser estimates of sphericity (ε = .71). We found a significant effect of condition, *F*_2.83, 67.86_ = 4.42, *p*<.01. Subsequently, we tested post-hoc for differences of VHI vividness scores in the incongruent conditions compared to the synchronous condition as well as for differences in the temporal order of visual and tactile events. There were significant differences for both the −600 and +600 condition compared to the synchronous 0 condition, *t*_24_ = −2.65 and *t*_24_ = −2.91; both *p*<.05, one-tailed. Further, there were significant differences between the −600 and −300 condition as well as the +600 and +300 condition, *t*_24_ = −2.29 and *t*_24_ = −2.35; both *p*<.05, one-tailed. However, we found no significant reductions in illusion vividness for either the −300 or the +300 condition compared to the synchronous 0 condition, *t*_24_ = −1.54; *p* = .14 and *t*_24_ = −.94; *p* = .36, one-tailed. Finally, there was also no significant difference between the −300 and the +300 condition (*t*_24_ = −.61, *p* = 1.00, two-tailed) or the −600 and +600 condition (*t*_24_ = −.03, *p* = 1.00, two-tailed).

### Results of fMRI data analyses

No neuronal activity survived whole brain correction in the regression analysis for illusory ownership experiences in the synchronous 0 condition. However, applying small volume correction revealed significant activity only in the right-hemispheric PMv (peak activity at x = 36, y = 10, z = 22; *Z* = 2.37; *p*<.01, uncorrected).

**Figure 4:**
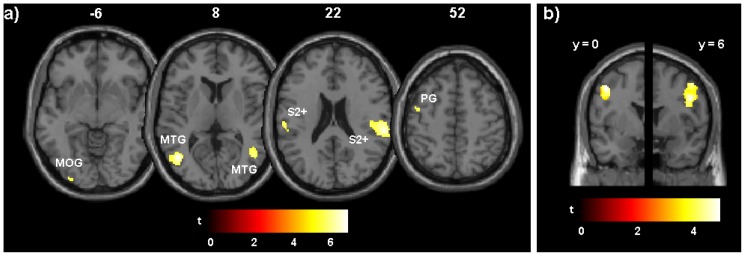
Results for the conjunction analysis. a) whole brain analysis (*p*<.05, FWE-corrected); the number above each slice indicates the height (z-coordinate in Montreal Neurological Institute [MNI] space); b) analysis for bilateral ventral premotor cortex using regions of interest (*p*<.005, uncorrected); given is the y-coordinate of the slices of peak activity (in MNI space). MOG =  middle occipital gyrus; MTG =  middle temporal gyrus; S2+ =  secondary somatosensory cortex plus its vicinity; PG =  precentral gyrus. Color bars indicate *t*-values.

The conjunction analysis across all conditions revealed bilateral activation in the middle temporal gyrus. Further, we found bilateral activity in a cluster reaching from the lower parts of the supramarginal gyrus to the upper parts of superior temporal gyrus, as well as a significant cluster in left middle occipital gyrus. Finally, significant activity in the left precentral gyrus was found (see Table 2 and Figure 4a). Applying ROIs reflecting the right and left ventral premotor cortices, we identified activity in the right (peak activity at x = 48, y = 6, z = 34; *Z* = 4.40; *p*<.005, FWE-corrected) and left PMv (peak activity at x = −44, y = 0, z = 46; *Z* = 4.69; *p*<.005, FWE-corrected). These clusters are displayed in Figure 4b. Applying ROIs reflecting the intraparietal cortex, we found significant activation only in the left hemisphere (peak activity at x = −34, y = −48, z = 50; *Z* = 3.68; *p*<.05, FWE-corrected).

As mentioned above, the analysis of the ratings indicated that both the −600 and +600 conditions – in contrast to both the -300 and +300 conditions – significantly diminish the VHI vividness scores compared to the synchronous stimulation. Accordingly, we contrasted the neuronal activity in the 0 condition against the neuronal activity in the combined ±300 and ±600 conditions. We found no significant activity in the 0>±300 contrast, neither in the whole brain analysis, nor the ROI analyses. This also holds for the inverse contrast. Although no activity survived the whole brain analysis in the 0>±600 contrast, the ROI analyses revealed activity in the right-hemispheric PMv (peak activity at x = 56, y = −6, z = 50; *Z* = 3.21; *p*<.01, FWE-corrected) and the left-hemispheric IPC (peak activity at x = −26, y = −56, z = 64; *Z* = 2.80; *p*<.005, uncorrected). There was no significant activation either for the PMv ipsilateral to stimulation site or the IPC contralateral to stimulation side. The inverse contrast (±600>0) did not reveal any significant activity, neither in the whole brain contrast nor by applying small volume correction. Finally, we performed contrasts for the temporal order of sensory events, but there were no significant activity differences, neither for the −600/−300>+600/+300 contrast, nor for the inverse contrast (this holds for the whole brain as well as for PMv and IPC analyses applying small volume correction).

## Discussion

The present study revealed first experimental data on the rubber hand illusion induced by a virtual reality set-up of an MRI compatible device. While previous studies already showed that participants can be induced to perceive ownership over an artificial limb [11], [14] or even an artificial body [17] in VR, studies applying functional imaging techniques used manual methods for illusion induction [3], [23], [24], [25], [31], [32], [33]. Compared to manual induction by a human experimenter, however, the present VR device allows a standardized induction of illusory ownership without social interaction or any inaccuracies regarding the application of the visuotactile stimulation and the position of the virtual limb.

The ratings show that the VHI in the present study was successfully induced by synchronous visuotactile stimulation. Temporal asynchronies between visual and tactile input reduced the vividness of illusory sensations in an approximately linear fashion, with longer delays resulting in lower scores of VHI vividness. Further, the decrease in illusion vividness was independent of the temporal order of the visual and tactile events.

Using fMRI, we found significant activation in bilateral PMv associated with the processing of visuotactile stimulation across all conditions. When the conditions of synchrony and distinct asynchrony were compared, we found significant PMv activity only contralateral to the stimulation site. This also applies to the regression analysis for the synchronous 0 condition which revealed specific activity in the right-hemispheric PMv associated with illusory limb ownership experiences. Further, we found stronger IPC activity ipsilateral to stimulation site associated with synchronous visuotactile stimulation.

In previous studies, the RHI was usually induced via stroking movements (e.g., [2], [3]). Due to the smooth on- and offset of stroking stimuli and their relatively long duration, this method warrants a high degree of spatiotemporal overlap between tactile and visual input, which facilitates perceiving them as being congruent. In the present study, we used considerably shorter, well-localized taps and demonstrated that they are equally appropriate for inducing illusory experiences. We found that synchronous touches induced a vivid VHI, as indicated by the participants' ratings. Illusory limb ownership was experienced in 28% of participants in the synchronous 0 condition, which is in line with the literature [23]. Although the participants reported weaker illusory sensations when there was a delay of 300 ms between visual and tactile input, significant reductions of VHI vividness scores were only reported when the delay was extended to 600 ms. However, there might be a generalized proneness to perceive illusory body ownership which is indicated by an intra-individually stable response pattern across all conditions: participants who scored high or low in the synchronous 0 condition tend to respond in a similar way to incongruent conditions. This finding is in line with the hypothesis of a stable perceptional trait to integrate body-related sensory input [23].

Our finding that perceived VHI vividness is significantly affected by distinct, but not slight asynchrony between tactile and visual stimulation replicates the results reported by Shimada et al. [7], suggesting that delays of 300 ms may reflect the temporal boundaries of visuotactile integration. This might suggest a defined breaking point in the mechanism for neuronally processed and perceived synchrony of sensory events. In the present study, we complemented these findings by fMRI data revealing the brain areas associated with sensory integration. Across each condition, we found activity in bilateral PMv, which has been shown to play a key role in coding the posture of one's own limbs. Previous studies showed that receptive fields in the PMv of primates code for the peripersonal space surrounding the body [34]. These receptive fields are formed by bimodal neurons responding to visual and proprioceptive input from the arms [35] or their close environment [28], [29], [35]. In accordance with these findings, illusory ownership for an artificial limb is associated with activity in the PMv [3], [24], [25], reflecting the important role of PMv in the integration of visual and somatosensory input. The general activation of the premotor cortex might also refer to functional properties such as sensory prediction and serial processing of stimuli, probably highlighting the contribution of the premotor cortex to the processing of sequentially structured sensory events [36].

Although we found the PMv to be bilaterally activated across conditions, the comparison between asynchronous and synchronous conditions, as well as the regression analysis for the synchronous 0 condition revealed activity only in the PMv contralateral to stimulation site. This finding might indicate an involvement of this area specifically in the integration of body-related sensory input applied to the contralateral limb as a necessary prerequisite for the experience of limb ownership [4]. The found precentral activity across all conditions might reflect additional effort of multisensory processing, since this region has been shown to support visoutactile integration [37].

Further, we found IPC activity only ipsilateral to stimulation site. The contralateral IPC has been found to be involved in the processing of synchronous visuotactile stimulation as well as the seen position of the artificial limb during the RHI paradigm [3]. Although its activity appears not to be directly associated with the experience of body ownership [3], parietal lesions have been shown to be associated with asomatognosia regarding one's own contralateral limb [38], probably due to its important role in integrating body-related sensory information [26], [39]. Recently, Brozzoli et al. [40] showed that IPC activity appears to reflect the felt position of a limb perceived as one's own in relation to close objects, highlighting its crucial role in coding the peri-hand space [29]. In the present study, we only found IPC activity ipsilateral to stimulation site, suggesting that there was no necessity to combine felt and seen position of one's own limb and its virtual counterpart. This might be related to our paradigm: in contrast to other studies using a rubber hand, we asked our participants to align their own hand to the seen hand in virtual reality. This had the consequence that both hands apparently shared the same space, minimizing the visuoproprioceptive incongruence of posture which is used to be solved by the IPC in the normal RHI paradigm, while simultaneously ensuring successful visuotactile integration [41]. The found IPC activity ipsilateral to stimulation side, however, might be related to rather unspecific processes of visual and somatosensory integration, which has been reported for the incorporation of objects not resembling a body part [42].

Finally, the subjective results as well as the fMRI data suggest that the temporal order of sensory events has no importance for illusion processing, neither for sensory integration nor for illusory sensations in the VHI paradigm. This finding may represent the high degree of generalization of the brain areas involved in multisensory integration: while there is a certain degree of discrepancy that is tolerated by integrative processes, the direction of this discrepancy is irrelevant.

Across all conditions, we found activation within the middle temporal gyrus (MTG). This general activity indicates that this area is not necessarily associated with the experience of illusory ownership, but rather with general features of illusion induction context. The MTG represents the junction between the occipital and the temporal cortex, and is comprised of areas involved in visual processing of objects and body parts, such as the extrastriate body area (EBA), the lateral occipital complex (LOC), and the middle temporal complex (MT+). The EBA has been shown to respond selectively to static or moving non-facial body parts [43], changes in limb position [44], and is involved in mental imagery of body parts [45], [46], and might even contribute to illusory limb ownership experiences [47]. Additionally, there might be an overlap of body part- and motion-selective responses in these lateral occipital areas [48]. However, since in the present study the virtual hand was present in both the on- and the off-blocks, the activity in MTG is less likely to reflect the EBA. This is also true for the LOC, the activity of which reflects higher-level shape processing [49]. The MT+ (consisting of the middle temporal and medial superior temporal areas [50]), however, specifically responds to visual motion [51], [52]. The involvement of the MT+ during VHI induction is plausible, since the visuotactile stimulation is necessarily associated with a moving object applying the tactile stimulation on the artificial limb. However, whether this MT+ activity – together with the found activity in middle occipital gyrus [53] – simply reflects attentional modulation in extrastriate visual cortex [54], [55], [56] due to perceived visuotactile synchrony [57], or whether it might indicate the earliest step necessary for illusory ownership experiences, remains open. Beauchamp et al. [58] suggested an involvement of subunits of the MT+ in eye-hand-coordination. Thus, its activity in the present study might contribute rather indirectly to illusory experiences by adapting to the recalibrated body coordinate systems caused by successful illusion induction [5].

Further, we found strong bilateral activity in an area including the superior temporal gyrus (STG) and the supramarginal gyrus (SMG). This activation resembles that reported by Beauchamp et al. [58], [59], who investigated the representation of single touches in somatosensory cortices. In accordance with these authors, we identified the region of activation in the present study as composed of the secondary somatosensory cortex (S2) and other somatosensory association areas such as the SMG. The SMG has been shown to be involved in the representation of the nearest peripersonal space of limbs in the human brain [60]. Further, it is a part in the neuronal network integrating visuotactile input applied to the hand [26], and other findings indicate that this region receives significant proprioceptive inputs [61]. S2 has been shown to be modulated by spatial attention [62], enhancing responses to tactile events. Additionally, touch observation as well as the experience of one's own body being touched activates S2 [63], [64].

## Conclusions

The present results elucidate the neuronal properties underlying sensory integration in the VHI paradigm. The brain demonstrates a certain flexibility to overcome temporal asynchrony between visual and tactile events as well as the temporal order of sensory stimuli. The induction of a vivid VHI through synchronous visuotactile stimulation was accompanied by PMv activity contralateral to stimulation site, emphasizing its role for the experience of illusory limb ownership. Finally, association areas such as the MTG and S2 seem to be involved in the processing of synchronous visuotactile input, probably reflecting necessary steps toward a conscious body perception through sensory integration.
